# Evaluation of the efficacy of topical cosmetic products in patients with hand-and-foot syndrome undergoing oncological treatments

**DOI:** 10.1093/oncolo/oyag233

**Published:** 2026-06-12

**Authors:** Antonia Martuscelli, Giulio Tosti, Patrick Maisonneuve, Carolina Redaelli, Mirella Indino, Martina Cereda, Giuseppe Curigliano, Ida Minchella

**Affiliations:** Oncology Aesthetics Center, IEO Istituto Europeo di Oncologia IRCCS, 20141 Milan, Italy; Dermato-Oncology Unit, IEO European Institute of Oncology IRCCS, 20141 Milan, Italy; Division of Epidemiology and Biostatistics, IEO European Institute of Oncology IRCCS, 20141 Milan, Italy; Oncology Aesthetics Center, IEO Istituto Europeo di Oncologia IRCCS, 20141 Milan, Italy; IEO Istituto Europeo di Oncologia IRCCS, 20141 Milan, Italy; IEO Istituto Europeo di Oncologia IRCCS, 20141 Milan, Italy; Department of Oncology and Hemato-Oncology, University of Milano La Statale, 20122 Milan, Italy; Division of Early Drug Development for Innovative Therapies, IEO European Institute of Oncology IRCCS, 20141 Milan, Italy; Division of Early Drug Development for Innovative Therapies, IEO European Institute of Oncology IRCCS, 20141 Milan, Italy

**Keywords:** hand-and-foot syndrome, skin toxicity, cosmetic treatment, quality of life

## Abstract

**Background:**

Hand–foot syndrome (HFS) is a complication of many anticancer therapies and negatively affects patients’ quality of life (QoL). Currently, little data are available on using non-pharmacological skin products as standard therapy for HFS.

**Aim:**

This study aims to investigate whether a specific set of cosmetic products is effective for managing HFS skin side effects in cancer patients.

**Materials and methods:**

This single-arm study involved 53 cancer patients with grade 1 HFS undergoing chemotherapy, targeted, or hormonal treatments at the European Institute of Oncology. The study included a baseline visit and a follow-up visit 45 days later. All enrolled patients were prescribed a set of four cosmetic products for skin treatment. Patients underwent instrumental measurement to evaluate skin hydration and erythema, while their QoL was assessed using the Skindex-16 self-questionnaire. Additionally, physicians clinically evaluated the HFS severity, using the CTCAE (Common Terminology Criteria for Adverse Events) scale and by comparing patient photographs.

**Results:**

No patients worsened. After 45 days, the application of the cosmetic set increased skin hydration by 33% (*P* < .0001), while skin erythema decreased by 82.9% (*P* < .0001). Patients also showed a significantly lower mean Skindex-16 score at 45 days (−35.5%, *P* < .0001) compared to baseline. These findings were associated with clinical improvement in HFS-related skin symptoms in 58.5% of patients.

**Conclusion:**

These results show that a set of cosmetic products specific for cancer skin care can effectively manage HFS-related symptoms, resulting in improved QoL for patients, regardless of the anticancer treatment received.

## Graphical Abstract

**Graphical Abstract:**
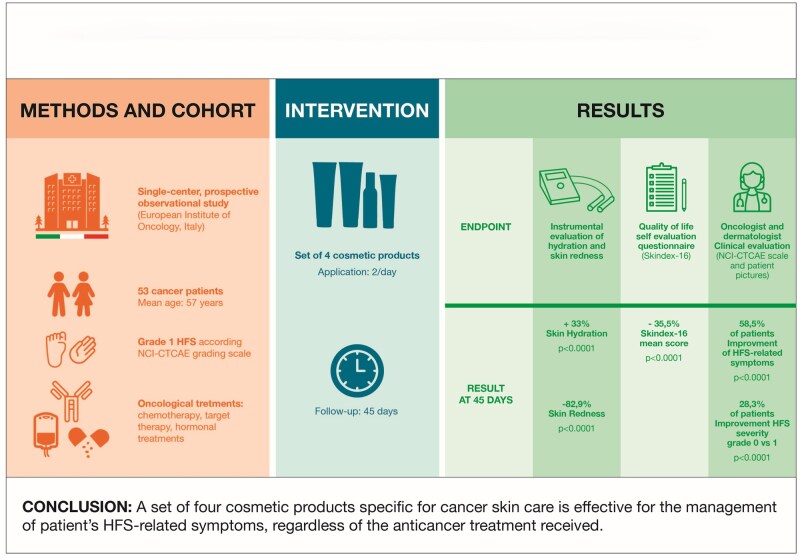

Implications for Practice Hand–foot syndrome (HFS) is a common skin toxicity associated with chemotherapy and cancer treatments. HFS can be managed through medications, such as topical steroids, as well as non-pharmacological strategies. This study explored using a specific set of cosmetic products to address HFS. The results showed that this non-pharmacological approach was effective from both clinical and patient perspectives, improving skin symptoms and quality of life without interrupting or modifying oncological treatment. These findings emphasize the importance of non-pharmacological products as supportive tools for healthcare professionals in managing skin toxicities in cancer patients

## Introduction

Current anticancer therapies have improved the prognosis and long-term survival for many malignancies, but they can all induce side effects, among which skin toxicity is particularly relevant.^1–6^

Hand–foot syndrome (HFS) is a common skin toxicity associated with many chemotherapeutic agents, newer targeted molecular therapies and some radiotherapies. At its onset, HFS presents with dysesthesia and paresthesia, tingling in the palms, fingers, and soles,^7^^,^^8^ which, without adequate management, may progress to burning pain, marked erythema with or without edema, skin desquamation, fissures, and ulceration.^8^ Several hypotheses about the mechanism of this syndrome have been advanced, but the actual mechanism remains unclear.^9^

Although HFS is typically self-limiting and rarely results in hospitalization, symptoms such as skin dryness and fissures can limit daily activities such as walking, holding objects, and performing simple tasks.^7^^,^^8^ This skin condition can significantly impair patient’s quality of life (QoL)^9–11^ and can lead to poor compliance with cancer treatment, delays in administration, or even interruption of the prescribed anticancer treatment.^12–15^

Several publications provide recommendations for pharmacological treatment, but non-pharmacological skincare still requires further exploration.^16^ Moreover, there is no established standard for preventing and managing the progression of skin toxicity caused by HFS. At present, the most effective way to managing HFS is by modifying the dose intensity or interrupting the treatment.^17^ High-potency topical corticosteroids can help reduce inflammation, and wound care can be provided for ulcerations.

The effectiveness of non-pharmaceutical products available for the prevention and treatment of HFS symptoms is generally less well-documented than that of pharmaceutical ones. Nevertheless, in recent years, an increasing number of studies have investigated the benefits of dermo cosmetic products, especially urea-based ones, for preventing this cutaneous toxicity, with variable results. Pyridoxine and uric acid–based cream do not show significant efficacy in clinical studies,^18^^,^^19^ while a randomized study in cancer patients treated with sorafenib demonstrated the activity of urea cream on cutaneous wetness.^20^ Various non-pharmaceutical products are also available for managing the overt symptoms of HFS: current approaches may include emollients, topical corticosteroids, and keratolytic agents, although more data are needed to assess their effectiveness and tolerability.^7^^,^^15^^,^^21–26^

For these reasons, the present study aims to evaluate whether a set of specific cosmetic products is effective for managing the HSF main skin side effects, such as xerosis and erythema, and improving patient’s QoL in a population of cancer patients undergoing different oncological treatments, considering instrumental measurements, patient’s subjective evaluation, and clinical evaluation.

Our previous study conducted with specific cosmetic products and aesthetic treatments showed effectiveness in managing the side effects of chemotherapy, target therapy, and radiotherapy on patient’s skin-related QoL (SRQoL) and distress.^27^

## Materials and methods

### Study design

This single-center, prospective, observational, single-arm study was conducted between September 2022 and November 2023 at the European Institute of Oncology (IEO), Milan, Italy.

The study was approved by the European Institute of Oncology Review Board and Ethics Committee (R1633/22-IEO 1748), and all participants provided written informed consent prior to taking part in the study. Participants were recruited consecutively from the oncology departments. The study involved two patient visits: a baseline visit and a follow-up visit, approximately 45 days later.

### Participants

Participants were diagnosed cancer patients receiving chemotherapy and targeted or hormonal treatments. Recruitment was performed by the medical oncologists based on the presence of HFS symptoms. The severity of HFS was graded according to the CTCAE v5.0 (Common Terminology Criteria for Adverse Events, version 5.0).^7^ The scale includes four grades: grade 0 (no signs), grade 1 (minimal skin changes without pain), grade 2 (skin changes with pain), and grade 3 (severe skin changes with pain, limiting activities of daily living). Only patients aged 18 or older, with a cancer diagnosis and presenting grade 1 adverse skin reaction, were enrolled. Patients with skin toxicities other than HFS or with HFS adverse skin symptoms higher than grade 1 were excluded. Additionally, patients with preexisting skin disorders that could interfere with the study results (such as dermatitis, psoriasis), those who were pregnant or breastfeeding or those with known hypersensitivity or allergy to any study product’s components were excluded from the study.

All enrolled patients were prescribed four cosmetic products (Ontherapy® by Dermophisiologique) for skin treatment: a cleansing cream, a soothing and nourishing cream, an anti-desquamation cream with urea 5%, and an emollient mixture. Box S1 provides a detailed description of the cosmetic properties and active principles.

### Outcomes

The primary outcome was to assess the effectiveness of the study products in reducing the severity of skin dryness in cancer patients after 45 days of use.

Secondary outcomes were the effectiveness of the cosmetic products in reducing the severity of erythema and in lessening the impact of the skin manifestations on patient QoL. This also included a clinical assessment conducted by the referring oncologist and dermatologist. At the end of the study, each patient completed a diary to assess compliance with the protocol.

### Intervention

The study included two clinical visits: baseline (T0, prior to starting the use of cosmetic products) and follow-up (T1, 45 days after starting the use of cosmetic products). Before enrollment, an oncologist examined the patient’s skin to assess eligibility criteria and to collect clinical data.

Measurements were carried out by an oncological esthetics specialist, a professional who specializes in protecting the skin from the toxic effects of oncological treatments. Before practicing, the oncological esthetics specialist undergoes a certified training program provided by the Professional Association of Oncological Esthetics.^27^^,^^28^

At the initial visit (T0), the oncologist assessed patients for adverse skin reactions and graded them based on the CTCAE v5.0 scale. Afterward, patients were directed to the IEO Oncology Esthetics Center, where the oncological esthetics specialist measured hydration and erythema in specific skin areas (the back and palms of the hands, the back and soles of the feet) and made a photographic report. Additionally, patients completed the Skindex-16 questionnaire, a self-reported QoL tool.

All patients received four commercially available cosmetic products (Ontherapy® by Dermophisiologique) formulated explicitly for cancer skincare. Patients were instructed to use each product following a specific protocol: in the morning, apply the affinity cleansing cream during skin cleansing, followed by the anti-desquamation cream; in the evening, apply affinity cleansing cream, followed by the application of the emollient mixture and then the soothing and nourishing cream. The date and time of each product application were then recorded in a diary to assess compliance.

At the follow-up visit (T1), the oncologist reassessed skin toxicity related to cancer treatment. Patients completed a follow-up Skindex-16 questionnaire, hydration and erythema measurements were recorded, and photographs were taken (in the same body areas analyzed during the first visit). At the end of the study, the oncologist and dermatologist evaluated the patient’s photographic record of HFS skin toxicity.

### Measures

Changes in skin hydration from baseline were assessed using the Corneometer® CM 825 probe, which analyses changes in the skin’s electrical capacitance related to water content.^29–32^ All measurements reported here represent the average of three assessments obtained on two skin areas: the dorsum of the right hand and the dorsum of the right foot.

Changes in skin erythema from baseline were measured photometrically using the Mexameter® MX 18 probe.^33–35^ All reported measurements are the average of three assessments obtained on four skin areas: the palm of the right hand, the fingertip of the right hand, the sole of the right foot, and the fingertip of the right foot.

Both measurements, using Corneometer® and Mexameter®, were carried out in the same room, under stable temperature and relative humidity conditions. Data were stored electronically using a laptop equipped with the appropriate software (Courage-Khazaka Electronics).

The impact of skin manifestations on patient QoL was evaluated using the Skindex-16,^36^ a 16-item self-report questionnaire previously used for patients with HFS.^37^^,^^38^ It is composed of three subscales: perceived symptoms (items 1-5), emotions (items 6-11), and daily functions (items 12-16). Responses to each item were transformed into a linear scale of 100, varying from 0 (never bothered) to 100 (corresponding to 6, always bothered). Each raw score was then normalized for statistical analysis. The final score represents the average of the patient’s responses within each domain.

The oncologist and dermatologist clinically evaluated HFS severity by comparing standardized patient photographs taken at baseline and at the end of the study in four body areas: the back of the hand, the back of the foot, the palm, and the sole.

The oncologist assessed changes in the degree of HFS toxicity using the CTCAEv5.0 scale.^7^ The dermatologist used a three-point evaluation (improvement, stability, and worsening) to assess the change in HFS-related skin symptoms (erythema, xerosis, and desquamation). Treatment benefit was evaluated based on the reduction in CTCAE grade and the improvement in skin symptoms.

The patient’s compliance with the product use protocol was assessed through a specific diary within 45 days, divided into morning and evening sections. Each entry included two tick boxes (YES for products applied or NO for products not applied), along with a NOTES section where the patient could indicate the reason for any missed application (Box S2).

### Sample size and statistical considerations

Anticipating similar results with the cosmetic protocol than those obtained with alternative moisturizing creams in the study by Di Franco et al.,^39^ we estimated that a sample of 48 patients would achieve 80% power to detect a difference of 3.6 between the null hypothesis (an increase in mean corneometry of 20 points corresponding to the minimal desired effect) and the alternative hypothesis (an increase of 23.6) with an estimated SD of 9.8 and with a significance level (alpha) of 0.05 using the one-tailed one-sample *t*-test.

Results were expressed as number and percentage or mean ± SD for categorical and continuous variables. Chi-square and Fisher’s exact tests were used to assess the differences in the distribution of categorical variables between subgroups of patients. Changes in quantitative parameters between baseline and 45 days were evaluated by the difference of mean values and 95% CIs obtained using a one-sample *t*-test. Variation of the mean difference between patient groups was assessed using analysis of variance (ANOVA). Graphical representation of the change of study outcomes (skin hydration, skin erythema, and Skindex-16 scales) for single patients and overall, between baseline and 45 days, was performed by combined box-and-whisker plots and spaghetti plots. All analyses were performed with the SAS software version 9.4 (Cary, NC, USA). Statistical significance was defined as two-tailed *P* < .05. Primary and secondary outcomes were measured at the end of the follow-up period in all enrolled subjects, regardless of the mode and completeness of exposure (intention to treat analysis [ITT]).

## Results

A total of 55 patients with grade 1 HFS were enrolled in the study. Two patients were excluded from the analysis due to hospital admission for health issues unrelated to the study. Therefore, data for 53 patients were included in the analysis.

Three categories of patients were defined based on the frequency of products used as follows: fully compliant patients applied the products each of the 45 days in the morning and in the evening (26.4%); mostly compliant omitted the products application on no more than ten occasions (43.4%), while less compliant omitted products application on more than ten occasions (30.2%). Patient demographics and clinical characteristics at baseline are listed in Table 1. Fifty-one (96.2%) of the 53 study participants were women. The median age was 57 years ranging from 27 to 78 years. Their most frequent diagnosis was breast cancer (*n* = 39). Most patients (48/53, 90.6%) were receiving cytotoxic chemotherapy alone or in combination with other treatments. Paclitaxel alone or in combination (60.4%) was the most frequently administered chemotherapeutic drug, followed by capecitabine and trastuzumab (20.8% each). About half (26, 49.1%) of the patients were initiating therapy when entering into the study, 22 (41.5%) were in the middle of their treatment cycle, and a minority (5, 9.4%) at the end of their cycle.

**Table 1:** Patient’s characteristics and compliance^a^ to the treatment.

	Total	Fully compliant no omission	Mostly compliant 1-10 omission	Less compliant >10 omissions	*P*-value
	Number (column %)	Number (row %)	Number (row %)	Number (row %)	
**All patients**	53 (100.0)	14 (26.4)	23 (43.4)	16 (30.2)	
**Age**					
** Median [range]**	57 [27-78]	60 [44-78]	57 [34-76]	57 [27-77]	
** <50 years**	8 (15.1)	1 (12.5)	4 (50.0)	3 (37.5)	
** 50-59 years**	24 (45.3)	6 (25.0)	10 (41.7)	8 (33.3)	
** ≥60 years**	21 (39.6)	7 (33.3)	9 (42.9)	5 (23.8)	.86
**Sex**					
** Female**	51 (96.2)	14 (27.5)	22 (43.1)	15 (29.4)	
** Male**	2 (3.8)	0 (0.0)	1 (50.0)	1 (50.0)	1.00
**Pathology**					
** Colon cancer**	2 (3.8)	0 (0.0)	2 (100.)	0 (0.0)	
** Breast cancer**	39 (73.6)	10 (25.6)	16 (41.0)	13 (33.3)	
** Ovarian cancer**	6 (11.3)	3 (50.0)	1 (16.7)	2 (33.3)	
** Lung cancer**	2 (3.8)	0 (0.0)	2 (100.)	0 (0.0)	
** Renal cancer**	4 (7.5)	1 (25.0)	2 (50.0)	1 (25.0)	.64
**Treatment^b^**					
** Chemotherapy**	48 (90.6)	13 (27.1)	20 (41.7)	15 (31.3)	.85
** Target therapy**	19 (35.8)	6 (31.6)	10 (52.6)	3 (15.8)	.23
** Immunotherapy**	4 (7.5)	0 (0.0)	2 (50.0)	2 (50.0)	.56
** Hormone therapy**	4 (7.5)	1 (25.0)	2 (50.0)	1 (25.0)	1.00
**Drugs^c^**					
** Capecitabine**	11 (20.8)	2 (18.2)	4 (36.4)	5 (45.5)	.52
** Carboplatin**	7 (13.2)	0 (0.0)	4 (57.1)	3 (42.9)	.29
** EC (epirubicin + cyclophosphamide)**	9 (17.0)	3 (33.3)	4 (44.4)	2 (22.2)	.90
** Paclitaxel**	32 (60.4)	9 (28.1)	13 (40.6)	10 (31.3)	.88
** Trastuzumab**	11 (20.8)	3 (27.3)	6 (54.5)	2 (18.2)	.63
**Cycle**					
** Start**	26 (49.1)	8 (30.8)	10 (38.5)	8 (30.8)	
** Middle**	22 (41.5)	6 (27.3)	10 (45.5)	6 (27.3)	
** End**	5 (9.4)	0 (0.0)	3 (60.0)	2 (40.0)	.76

a Compliance: the number of applications reported by the patients in their diary: fully compliant (no omission in product application), mostly compliant (no more than 10 omissions in product application), less compliant (more than 10 omission in product application).

b A patient may have received more than one therapy.

c Only drugs administered to five or more patients are reported in the table.

### Change in skin hydration and erythema at 45 days

Both skin hydration and skin erythema improved significantly after the intervention.

**Figure 1.**
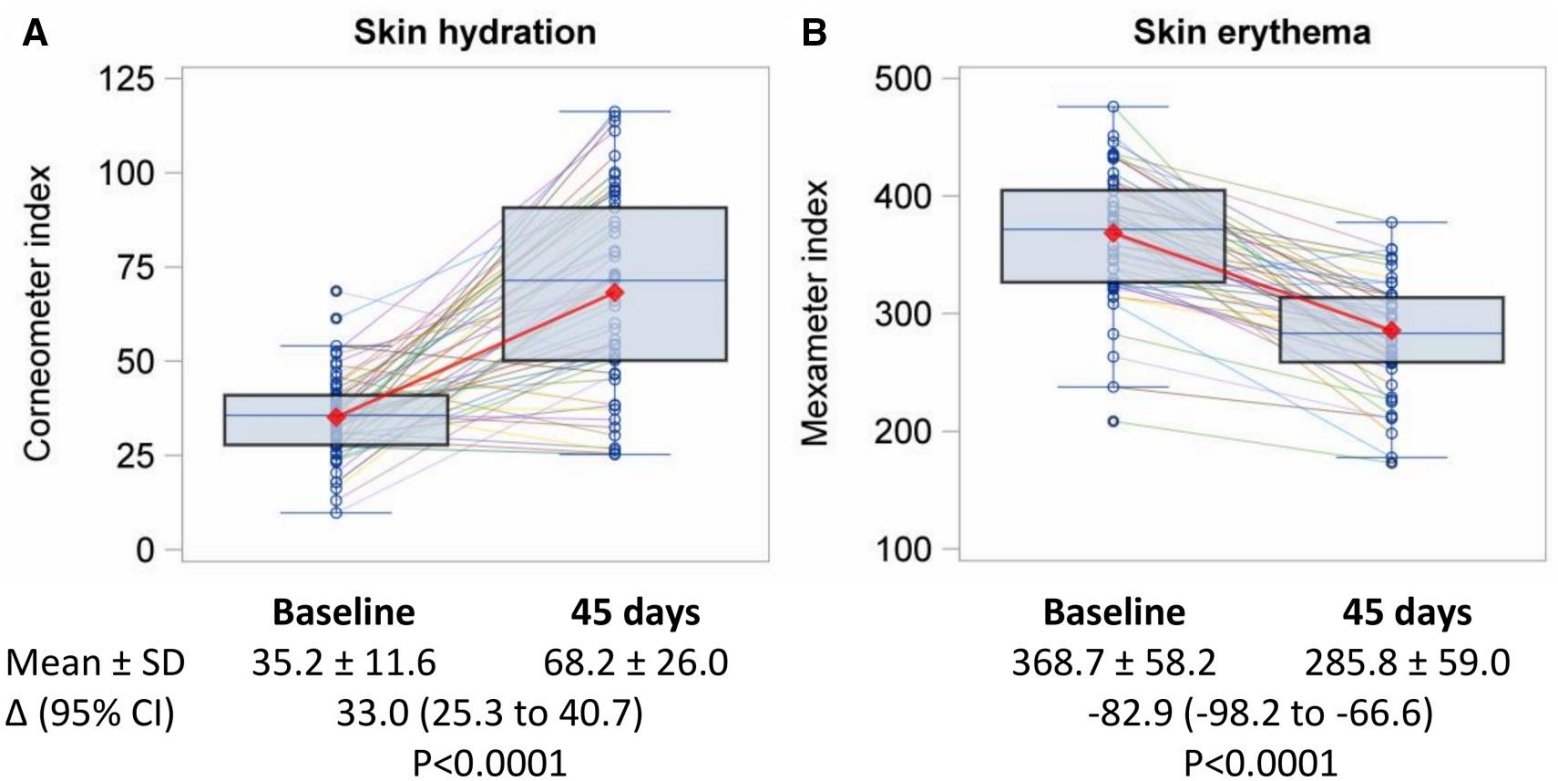
Instrumental change in skin hydration and skin erythema. (A) Change in skin hydration assessed by Corneometer® in the whole population of patients. (B) Change in skin erythema assessed by Mexameter® in the whole population of patients. The diagram labels report mean scores of skin hydration and skin erythema at T0 (baseline) and T1 (45 days, follow-up). A thick line connects the mean scores. Δ, mean difference.

At baseline, the mean skin hydration index assessed with the Corneometer® was 35.2 ± 11.6 with no differences across age groups, sex, type of cancer, or treatment cycle (Table S1). The mean hydration index was calculated as the average of three consecutive measures taken at multiple sites: the back of the right hand and the dorsum of the right foot. After 45 days, the Corneometer® measure was 68.2 ± 26.0, corresponding to an increase in hydration of 33.0 points (Table 2, Figure 1A).

**Table 2:** Skin hydration, skin erythema, and Skindex-16 mean scores at T0 and T1.

Measure	T0 baseline mean ± SD	T1 follow-up (45 days)
		mean ± SD
**Corneometer^®^ skin hydration^a^**	35.2 ± 11.6	68.2 ± 26.0
**Mexameter^®^ skin erythema^a^**	368.7 ± 58.2	285.8 ± 59.0
**Skindex-16 symptoms score**	60.0 ± 24.0	19.7 ± 19.9
**Skindex-16 functioning score**	43.5 ± 21.0	15.0 ± 16.8
**Skindex-16 emotions score**	54.4 ± 20.4	16.8 ± 18.9

a Average of three consecutive measures at multiple sites: back of the right hand and dorsum of the right foot for hydration, right-foot plant, right-foot fingertip, right-hand palm, and right-hand fingerprint for erythema.

At baseline, the mean skin erythema index measured by the Mexameter® was 368.7, with no differences across age groups, sex, or treatment cycle (Table S1). The mean erythema index was calculated as the average of three consecutive measures at multiple sites: right-foot plant, right-foot fingertip, right-hand palm, and right-hand fingertip. After 45 days, the Mexameter® measure was 285.8 ± 59.0, corresponding to a decrease in erythema of −82.9 points (Table 2, Figure 1B).

### Change in Skindex-16 at 45 days

**Figure 2.**
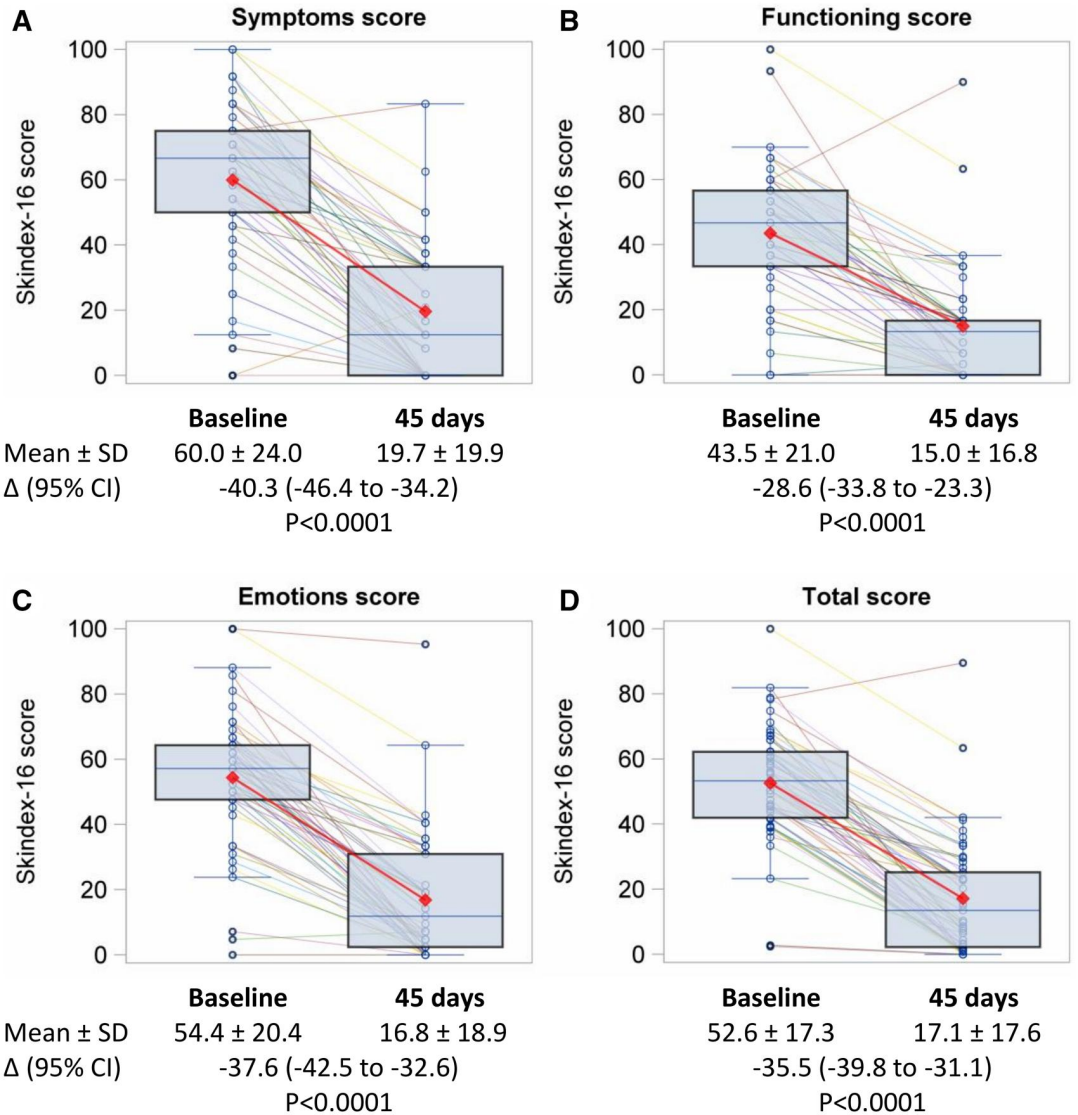
Change in Skindex-16 scale scores. Interaction between time and cosmetic treatment on perceived symptoms (A), functioning (B), and emotion (C) in the whole patient population. The diagram labels report the mean scores of the Skindex symptoms subscale at T0 and T1. A thick line connects the mean scores. (D) Total score represents the average of the three subscale scores. Δ, mean scores.

All patients completed the Skindex-16 questionnaire at baseline and after 45 days to evaluate the impact of skin manifestation on QoL. The study population at 45 days follow-up had a mean overall Skindex-16 score of 17.1 ± 17.6, which was lower than the baseline of 52.6 ± 17.3 (*P* < .0001). The total Skindex-16 score corresponds to the average of the three subscales (Figure 2D). We then investigated the trends of the three Skindex-16 subscales (ie, perceived symptoms, functioning, and emotions). At baseline, there was no difference across the type of cancer, treatment cycle, or level of compliance (Table S1).

Results at 45 days showed an improvement in each subscale from baseline to follow-up: symptoms domain value decreased from 60.0 ± 24.0 to 19.7 ± 19.9 (*P* < .0001, Figure 2A), function decreased from 43.5 ± 21.0 to 15.0 ± 16.8 (*P* < .0001, Figure 2B), and emotions from 54.5 ± 20.4 to 16.8 ± 18.9 (*P* < .0001, Figure 2C). The mean scores for the Skindex subscales over time are reported in Table 2. Additionally, the change in every single item of the questionnaire, scored from 0 (never bothered) to 6 (always bothered), is reported in Figure S1.

### Clinical improvement

At 45 days, the grade of HFS based on the CTCAE v5.0 classification, evaluated by the referring oncologist, regressed from grade 1 (minimal skin changes without pain) to grade 0 (no signs) in 15 patients, corresponding to 28.3%.

The dermatologist and medical oncologist assessed the clinical evaluation based on a review of photographs taken before and after intervention (T1 vs T0) of four body areas: the back of the hand, the back of the foot, the palm, and the sole. An improvement in HFS-related skin symptoms (including erythema, xerosis, and desquamation) was observed in 31 patients (58.5%).

**Figure 3.**
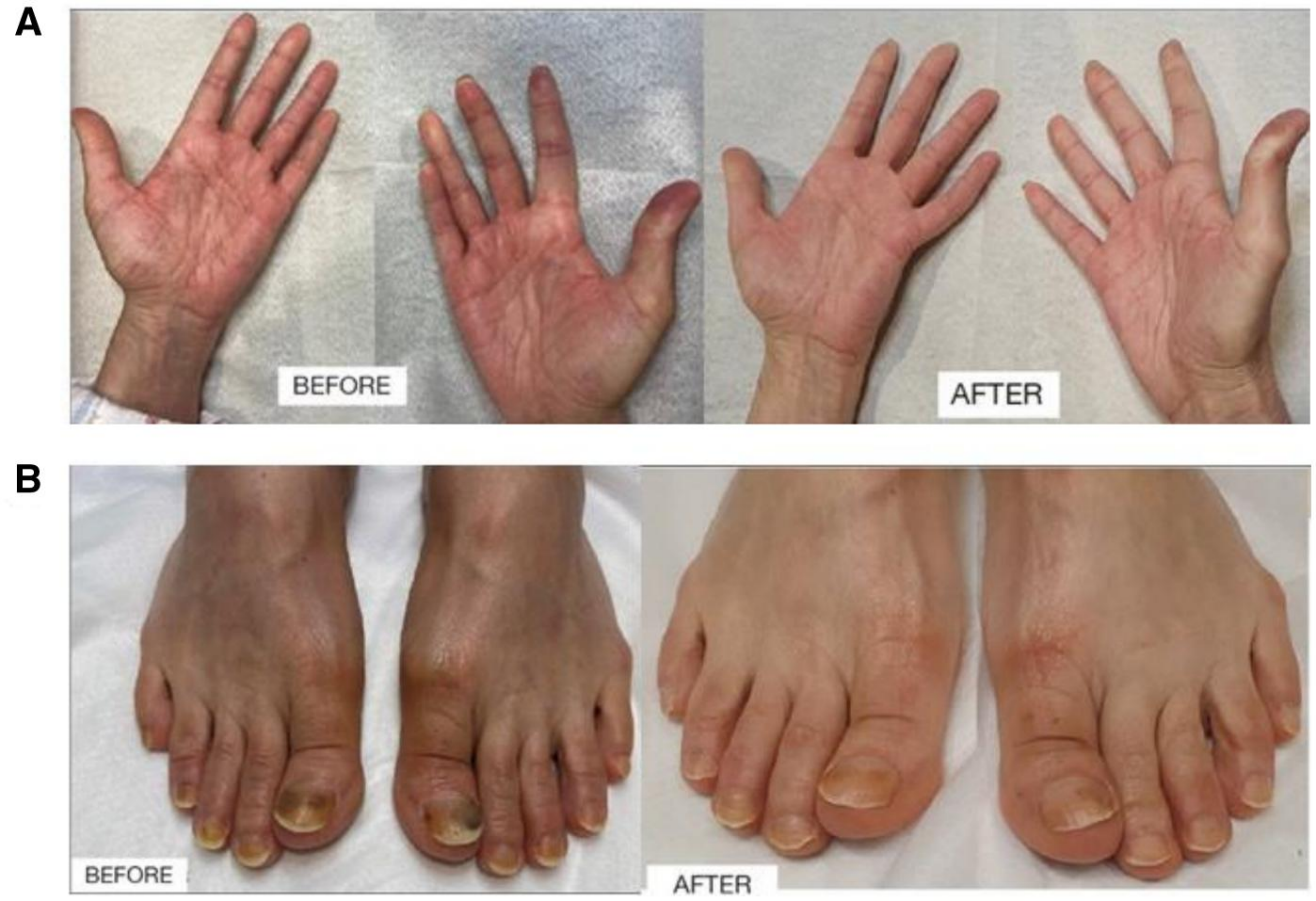
Hand-foot syndrome before (T0) and after (T1) application of topical cosmetic products. (A) Hand–foot syndrome symptoms of desquamation and erythema reduction in the hand palm of patient number 22 with metastatic breast cancer under treatment with chemotherapy. (B) Hand–foot syndrome symptoms of desquamation and erythema reduction in the back of the foot of patient number 38 with breast cancer under treatment with chemotherapy.

As for other outcomes, clinical improvement did not vary across patient subgroups (Table S2). Representative photographs taken before and after the intervention are shown in Figure 3.

At the end of the study, the investigating physicians rated their opinion regarding the skin benefit of the cosmetic treatment protocol as being good. In detail, the 15 patients with CTCAE grade improvement also showed clinical improvement.

No patient experienced worsening of symptoms to CTCAE grade 2 or grade 3, and all analyzed patients were able to pursue and complete their anticancer treatment.

## Discussion

Most currently available anticancer therapies are associated with uncomfortable skin reactions, such as HFS, which can cause significant discomfort and impair function, thereby affecting patient’s QoL and adherence to treatment.^40^

The use of non-pharmaceutical products to manage HFS symptoms is generally less well-documented than pharmaceutical approaches. Pyridoxine has not demonstrated superiority over placebo in either the prevention or treatment of HFS in patients receiving capecitabine.^41^^,^^42^ This lack of efficacy has also been confirmed by a recent meta-analysis in which pyridoxine was not able to prevent capecitabine-induced HFS.^19^ Some studies have evaluated prophylactic treatments for HFS using 10% urea-based topical products with variable outcomes. Wolf et al. compared the preventative use of a urea/lactic acid topical agent (ULABTKA), versus a placebo cream in 137 randomized cancer patients, and found that the use of ULABTKA did not significantly alter the proportion of patients presenting with moderate/severe HFS symptoms between the two groups.^18^ In contrast, Lien et al. show that using a urea-based cream twice daily prophylactically reduces HFS incidence compared to moisturizer alone in patients with hepatocellular carcinoma treated with sorafenib.^43^ Hofheinz et al. compared the prophylactic use of a urea-based cream and a medical ointment called Mapisal during the first 6 weeks of capecitabine treatment in 152 randomized cancer patients, demonstrating that the application of urea cream is an effective strategy for preventing HFS.^44^

Few products other than urea-based ones, and without keratolytic action, have been studied in the management of established HFS symptoms, rather than for their prevention.

In this study, 53 patients with various malignancies and grade 1 HFS, undergoing different anti-cancer therapies, were prescribed a non-pharmaceutical skin care set (Ontherapy® by Dermophisiologique) without concurrent medications. This allowed for precise evaluation of the product’s efficacy as a standalone treatment for HFS-related skin toxicity.

The primary finding of this observational, single-arm study is that both skin hydration and erythema significantly improved following daily use of the non-pharmaceutical skincare under real-life conditions. This is particularly important, as dryness and redness are common symptoms which, if not appropriately managed, may progress to cracking, blistering, or even ulceration, further impairing daily function and increasing infection risk.^45–47^ A significant improvement was also observed in patient’s QoL, as measured by the Skindex-16 self-assessment tool after 45 days of treatment. This is a crucial finding, as self-reported assessments provide direct insight into the patient’s experience, capturing aspects of well-being that may not be evident through clinical or instrumental means.

Importantly, improvements were observed across all domains of the Skindex-16: perceived symptoms, emotional impact, and social/functional impairment.

These findings were also supported by overall clinical evaluations from the investigating physicians, who noted that all patients showing improvement in HFS severity according to the CTCAE scale also demonstrated clinical benefit as evidenced by photographic documentation.

It is also noteworthy that the improvements in dermatological symptoms and QoL were observed across all types of anticancer therapy, underscoring the generalizability of the findings, regardless of the treatment received.

Despite these promising results, the single-arm design of the study presents inherent limitations. Chief among these is the absence of a control group, which makes it difficult to attribute the observed benefits to the skincare protocol alone definitively. Without comparison to either an untreated group or one treated with a placebo vehicle, it is challenging to determine whether the outcomes were directly due to the intervention or potentially influenced by uncontrolled external variables. Nonetheless, in an oncology setting where skin toxicities may compromise adherence to treatment, findings from uncontrolled studies may still hold significant clinical relevance. The implementation of a specific dermo cosmetic protocol, despite involving direct costs, could offer considerable indirect benefits, such as improved treatment adherence and better QoL, key objectives in supportive cancer care.

The open-label nature of the study may also introduce potential biases in both observation and interpretation of the data by clinicians and patients, since all participants were aware of the treatment being administered. To mitigate such biases, several strategies were employed: validated tools were used to assess cutaneous toxicity (CTCAE scale) and patient-reported QoL (Skindex-16), thereby ensuring standardized and reliable evaluations; objective endpoints were also included, using validated instruments to measure skin-related parameters. Furthermore, clinical assessments were not influenced by either instrumental results or patient feedback, as oncologists and dermatologists were blinded to the findings of the other evaluation methods before and after treatment. This strengthens the reliability of the outcomes, as the convergence of clinical, subjective, and instrumental evidence suggests a genuine and meaningful improvement.

It should also be noted that this study only assessed the effectiveness of the topical cosmetic protocol in managing HFS symptoms over a relatively short duration (45 days). Thus, it remains unclear whether the observed improvements in QoL and skin symptoms can be sustained over the long term.

Future studies should aim to replicate these preliminary results, to identify the most effective non-pharmacological topical products for HFS management and develop evidence-based guidelines for integrating dermo cosmetic interventions into oncological clinical practice.

Despite its limitations, this pre–post analysis has several strengths that support the validity of the findings. All patients completed the treatment, and no withdrawals due to adverse effects were reported, indicating that the topical cosmetic protocol was well tolerated and acceptable to patients. The high completion rate enabled the collection of both objective and subjective data, suggesting that cosmetic products may be a viable option not only for preventing the onset of HFS symptoms but also for treating existing symptoms, ultimately enhancing patient compliance with anticancer therapy.

## Conclusions

Our study indicates that non-pharmacological interventions, using a set of four cosmetic products developed explicitly for oncology-related skin care, can effectively manage HFS symptoms, significantly reducing dryness and skin erythema, regardless of the type of anticancer therapy. These improvements in objectively measured clinical signs were mirrored by enhanced patient-reported QoL and confirmed by clinical evaluations from oncologists and dermatologists.

In the management of HFS in cancer patients, the role of the oncological esthetics specialist is pivotal, operating in close collaboration with physicians. This specialized professional brings valuable expertise in non-pharmacological treatments, particularly the use of cosmetic products tailored for oncology skin care and their partnership with oncologists ensures a holistic approach that considers both cancer therapy and skin health.

Although additional studies are required to evaluate the long-term benefits and cost effectiveness, our findings support the use of suitable cosmetic products as part of complementary care for managing HFS side effects, and they highlight the necessity of further research in this domain.

## Supplementary Material

oyag233_Supplementary_Data

## Data Availability

The data underlying this article are available in the article and in its online supplementary material. Study details can be found with ClinicalTrials.gov Identifier: NCT06586073 at www.clinicaltrials.gov.
