# Supplementary material for: Evaluation of the efficacy of topical cosmetic products in patients with hand-and-foot syndrome undergoing oncological treatments
Source: Oncologist. 2026 Jun 12;31(8):oyag233. doi: 10.1093/oncolo/oyag233 (PMC13351728; doi:10.1093/oncolo/oyag233)
Supplement: oyag233_Supplementary_Data [file oyag233_supplementary_data.zip › Supplement_Data/Supplementary Box S1.docx]

Original Article

**Evaluation of the efficacy of topical cosmetic products in patients with hand-and-foot syndrome (HFS) undergoing oncological treatments**

Antonia Martuscelli, MSc^1*^; Giulio Tosti, MD^2^; Patrick Maisonneuve, DiplEng^3^; Carolina Redaelli, MD^1^; Mirella Indino^4^; Martina Cereda^4^; Giuseppe Curigliano, MD, PhD^5,6^; Ida Minchella, MD^6^

^1^Oncology Aesthetics Center, IEO Istituto Europeo di Oncologia IRCCS, 20141 Milan, Italy

^2^Dermato-Oncology Unit, IEO European Institute of Oncology IRCCS, 20141 Milan, Italy

^3^Division of Epidemiology and Biostatistics, IEO European Institute of Oncology IRCCS, 20141 Milan, Italy

^4^IEO Istituto Europeo di Oncologia IRCCS, 20141 Milan, Italy

^5^Department of Oncology and Hemato-Oncology, University of Milano La Statale, 20122 Milan, Italy

^6^Division of Early Drug Development for Innovative Therapies, IEO European Institute of Oncology IRCCS, 20141 Milan, Italy

*** Corresponding author**: Antonia Martuscelli. M.Sc; Scientific coordinator at Oncology Aesthetics Center, IEO. Address: IEO Istituto Europeo di Oncologia IRCCS, via Ripamonti 435, 20141, Milan, Italy. E-mail: [a.martuscelliresearch@dermophisiologique.it](mailto:a.martuscelliresearch@dermophisiologique.it) .

**SUPPLEMENTARY MATERIALS**

**Supplementary Box S1: Description of cosmetic products, active principles, and therapeutic properties**

**ONTHERAPY^®^ NORMALIZING-PROTECTIVE CLEANSING CREAM**

This non-foaming detergent emulsion cleanses the skin without altering its natural hydrolipidic film thanks to a selected mix of lipids and plant derived. It also contains natural substances such as Chamomile extracts, Hamamelis Virginiana and Malva Sylvestris which have a soothing action.


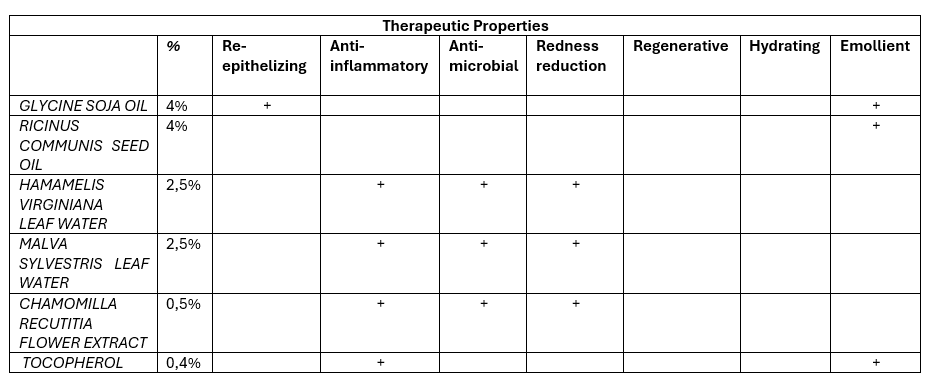
It can be used on the body and face when the skin is extremely dry and presents redness, itching and flaking.

SOURCES: Lin (2017), Pinto (2022), Amendola (2024), Piazza (2022), Yarijani (2018), Prudente (2017), Lairikyengbam (2024), Yamamoto (2010), Duman (2011), Gialaim (2023)

**ONTHERAPY^®^ ANTI-DESQUAMATION CREAM WITH UREA 5%**

The emulsion has hydrating and restorative properties specific for hands, feet, nails and heavily flaking areas. The balanced formula in 5% urea and allantoin, with a hydrating and re-epithelizing action guarantees the rebalancing of skin flaking, without keratolytic action, thus avoiding skin discomfort such as burning and irritation. The presence of emollient lipids such as Jojoba Oil, Rice and Bran oil, together with hyaluronic acid makes this product suitable for the daily treatment of particularly chapped and flaky skin, guaranteeing softness and deep hydration to the skin.


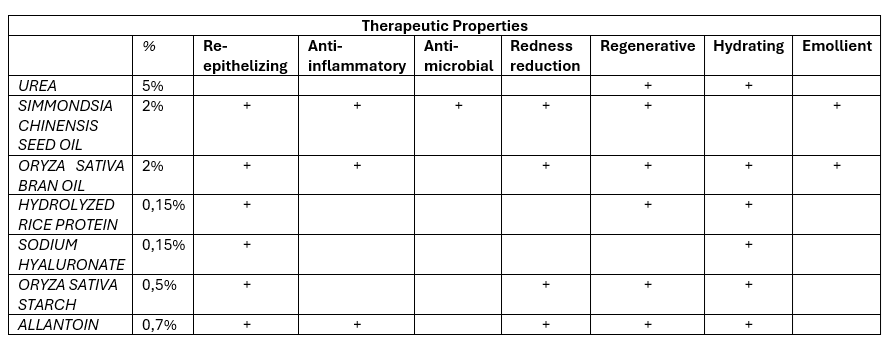
SOURCES: Lan (2022), Gialaim (2023), Piquero-Casals (2021), Gad (2021), Sabu (2022), Expand (2022), Al-Halaseh (2022), Burlando (2014), Fabrocini (2013), Czajkowska-Kośnik (2023), Saucedo-Acuña (2023)

**ONTHERAPY^®^ EMOLLIENT OIL**

A blend of vegetable oils and butters (sweet almond oil, jojoba oil, sunflower seed oil, shea butter) balanced to imitate the lipid composition of the skin surface to obtain an emollient, hydrating and protective effect on the skin. The presence of tocopherol gives an antioxidant and regenerating action.


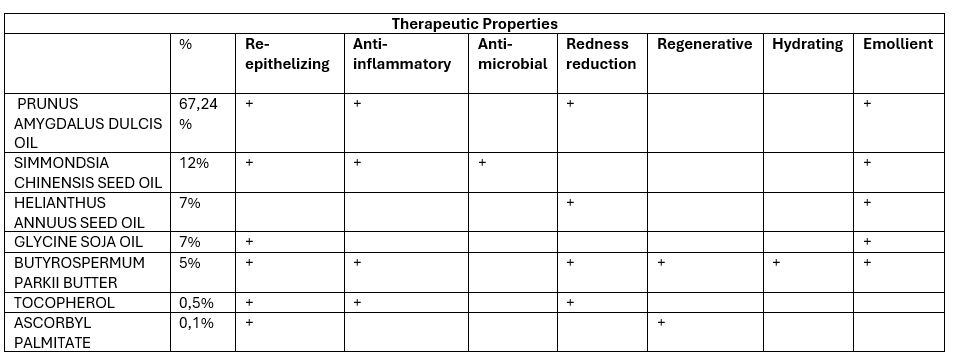
The product suits all situations where barrier alterations, excessive dryness, redness and itching occur.

SOURCES: Ouzir (2021), Gad (2021), Bashir (2021), Kumar (2022), Lin (2017), Ugwu‐Dike (2022), Ayanlowo (2022), Dreno (2023), Yamamoto (2010), Duman (2011), Gialaim (2023), Weeks (2023), Al-Niaimi (2017)

**ONTHERAPY^®^ SOOTHING AND NOURISHING CREAM**


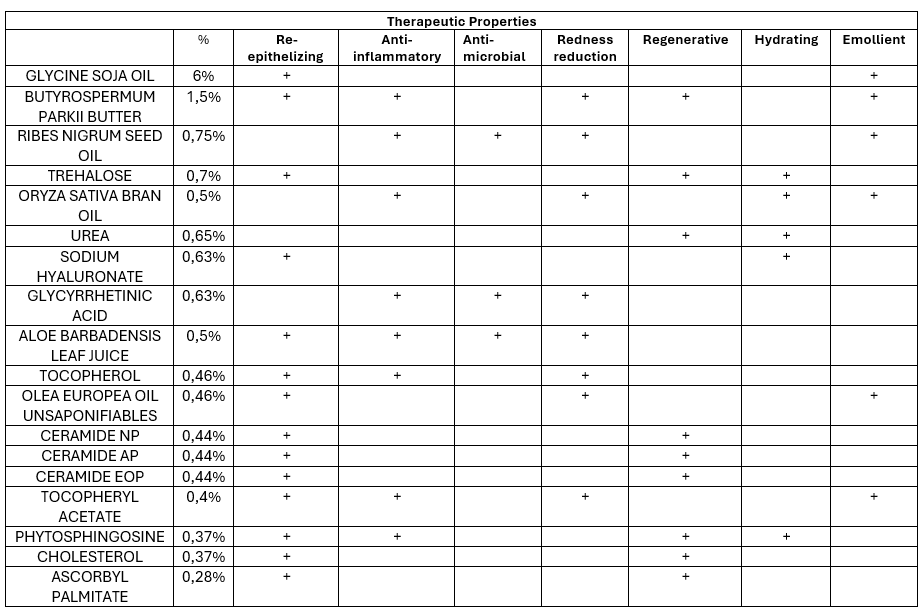
An emulsion with liquid-crystal technology, characterized by a rich and smooth texture, ideal for face and body. It is enriched in plant extracts such as black currant oil, 18β glycyrrhetinic acid and aloe, shown in the literature to display several properties, including soothing and anti-inflammatory activities to reduce redness and irritation of the skin. The formulation also contains shea butter, olive oil, ceramides and sterols that, in addition to reducing trans epidermal water loss, can replenish the skin’s lipid composition and strengthen its barrier function. Hyaluronic acid and urea are helpful to maintain the right level of hydration of the skin. It should be used on dehydrated skin, with redness and itchiness.

SOURCES: Lin (2017), Ugwu-Dike (2022), Ayanlowo (2022), Dreno (2023), Oczkowski (2021), Kendir (2016), Kupnik (2023), Sabu (2022), Expand (2022), Lan (2022), Gialaim (2023), Piquero-Casals (2021), Al-Halaseh (2022), Richard (2021), Yang (2020), Naini (2021), Govindarajan (2020), Donato-Trancoso (2023), Dauber (2023), Uchida (2021), Kahraman (2019), Nădăban (2023), Sochorová (2019), Yamamoto (2010), Duman (2011), Gialaim (2023), Weeks (2023), Al-Niaimi (2017)

**REFERENCES**

Lin, T. K., Zhong, L., & Santiago, J. L. (2017). Anti-inflammatory and skin barrier repair effects of topical application of some plant oils. *International journal of molecular sciences*, *19*(1), 70.

Pinto, J. R., Monteiro e Silva, S. A., Holsback, V. D. S. S., & Leonardi, G. R. (2022). Skin occlusive performance: Sustainable alternatives for petrolatum in skincare formulations. *Journal of Cosmetic Dermatology*, *21*(10), 4775-4780.

Amêndola, I., Viegas, D. D. J., Freitas, E. T., Oliveira, J. R. D., SANTOS, J. G. D., Oliveira, F. E. D., ... & Back-Brito, G. N. (2024). Hamamelis virginiana L. extract presents antimicrobial and antibiofilm effects, absence of cytotoxicity, anti-inflammatory action, and potential to fight infections through the nitric oxide production by macrophages. *Anais da Academia Brasileira de Ciências*, *96*(1), e20200031.

Piazza, S., Martinelli, G., Magnavacca, A., Fumagalli, M., Pozzoli, C., Terno, M., ... & Sangiovanni, E. (2022). Unveiling the ability of witch hazel (Hamamelis virginiana L.) bark extract to impair keratinocyte inflammatory cascade typical of atopic eczema. *International journal of molecular sciences*, *23*(16), 9279.

Yarijani, Z. M., Godini, A., Madani, S. H., & Najafi, H. (2018). Reduction of cisplatin-induced renal and hepatic side effects in rat through antioxidative and anti-inflammatory properties of Malva sylvestris L. extract. *Biomedicine & Pharmacotherapy*, *106*, 1767-1774.

Prudente, A. S., Sponchiado, G., Mendes, D. A., Soley, B. S., Cabrini, D. A., & Otuki, M. F. (2017). Pre-clinical efficacy assessment of Malva sylvestris on chronic skin inflammation. *Biomedicine & Pharmacotherapy*, *93*, 852-860.

Lairikyengbam, D., Wetterauer, B., Schmiech, M., Jahraus, B., Kirchgessner, H., Wetterauer, P., ... & Samstag, Y. (2024). Comparative analysis of whole plant, flower and root extracts of Chamomilla recutita L. and characteristic pure compounds reveals differential anti-inflammatory effects on human T cells. *Frontiers in Immunology*, *15*, 1388962.

Yamamoto, D., Yamamoto, C., Iwase, S., Kuroda, Y., Odagiri, H., & Nagumo, Y. (2010). Efficacy of vitamin E treatment for hand-foot syndrome in patients receiving capecitabine. *Breast Care*, *5*(6).

Duman, B. B., Kara, B., Kara, I. O., Demiryurek, H., & Aksungur, E. (2011). Hand-foot syndrome due to sorafenib in hepatocellular carcinoma treated with vitamin E without dose modification; a preliminary clinical study. *J Balk Union Oncol*, *16*(4), 759-764.

Lan, T. C., Tsou, P. H., Tam, K. W., & Huang, T. W. (2022). Effect of urea cream on hand-foot syndrome in patients receiving chemotherapy: a meta-analysis. *Cancer nursing*, *45*(5), 378-386.

Gialaim Purcino dos Reis, F. C., Menêses, A. G. D., Mazoni, S. R., Pereira Silveira, R. C. D. C., Diniz dos Reis, P. E., & Vasques, C. I. (2023). Topical interventions for preventing hand-foot syndrome resulting from antineoplastic therapy: A scoping review. *Revista da Escola de Enfermagem da USP*, *57*, e20220107.

Piquero-Casals, J., Morgado-Carrasco, D., Granger, C., Trullàs, C., Jesús-Silva, A., & Krutmann, J. (2021). Urea in dermatology: a review of its emollient, moisturizing, keratolytic, skin barrier enhancing and antimicrobial properties. *Dermatology and therapy*, 1-11.

Gad, H. A., Roberts, A., Hamzi, S. H., Gad, H. A., Touiss, I., Altyar, A. E., ... & Ashour, M. L. (2021). Jojoba Oil: An updated comprehensive review on chemistry, pharmaceutical uses, and toxicity. *Polymers*, *13*(11), 1711.

Sabu, V., & Helen, A. (2022). Njavara rice (Oryza sativa Linn.) bran oil exerts anti-inflammatory effects through regulation of Notch-mediated T-cell receptor (TCR) activation in experimentally induced atherosclerosis. *Cellular and Molecular Biology*, *68*(10), 21-29.

Expand, C., Care, D. S., Barrier, P. Y. S., & Card, G. Benefits of Rice Bran Oil for Dry Skin.

Yu, Y., Gaine, G. K., Zhou, L., Zhang, J., Wang, J., & Sun, B. (2022). The classical and potential novel healthy functions of rice bran protein and its hydrolysates. *Critical reviews in food science and nutrition*, *62*(30), 8454-8466.

Al-Halaseh, L. K., Al-Jawabri, N. A., Tarawneh, S. K., Al-Qdah, W. K., Abu-Hajleh, M. N., Al-Samydai, A. M., & Ahmed, M. A. (2022). A review of the cosmetic use and potentially therapeutic importance of hyaluronic acid. *Journal of Applied Pharmaceutical Science*, *12*(7), 034-041.

Fabrocini, G., Izzo, R., Panariello, L., & Monfrecola, G. (2013). Skin reactions secondary to anticancer agents. *Eur Med J Dermatol*, *1*, 38-43.

CZAJKOWSKA-KOŚNIK, A. N. N. A., BAGIŃSKA, Z. H., & WINNICKA, K. (2023). EMULSIONS CONTAINING ALLANTOIN AND D-PANTHENOL AS ATTRACTIVE MOISTURIZING AGENTS. *Acta Poloniae Pharmaceutica*, *80*(6).

Saucedo-Acuña, R. A., Meza-Valle, K. Z., Cuevas-González, J. C., Ordoñez-Casanova, E. G., Castellanos-García, M. I., Zaragoza-Contreras, E. A., & Tamayo-Pérez, G. F. (2023). Characterization and in vivo assay of allantoin-enriched pectin hydrogel for the treatment of skin wounds. *International Journal of Molecular Sciences*, *24*(8), 7377.

Ouzir, M., Bernoussi, S. E., Tabyaoui, M., & Taghzouti, K. (2021). Almond oil: A comprehensive review of chemical composition, extraction methods, preservation conditions, potential health benefits, and safety. *Comprehensive reviews in food science and food safety*, *20*(4), 3344-3387.

Bashir, T., Zia-Ur-Rehman Mashwani, K. Z., Haider, S., & Shaista Tabassum, M. (2021). 02. Chemistry, pharmacology and ethnomedicinal uses of Helianthus annuus (Sunflower): A Review. *Pure and Applied Biology (PAB)*, *4*(2), 226-235.

Kumar, V., Kumar, A., Mishra, S., Kan, P., Ashraf, S., Singh, S., ... & Shivgarh Emollient Research Group. (2022). Effects of emollient therapy with sunflower seed oil on neonatal growth and morbidity in Uttar Pradesh, India: a cluster-randomized, open-label, controlled trial. *The American Journal of Clinical Nutrition*, *115*(4), 1092-1104.

Ugwu‐Dike, P., & Nambudiri, V. E. (2022). A review of ethnomedicinal uses of shea butter for dermatoses in sub‐saharan africa. *Dermatologic Therapy*, *35*(3), e14786.

Weeks, B. S., Fu, R., & Zaidi, M. (2023). Vitamin C Promotes Wound Healing: The Use of In Vitro Scratch Assays to Assess Re-Epithelialization.

Al-Niaimi, F., & Chiang, N. Y. Z. (2017). Topical vitamin C and the skin: mechanisms of action and clinical applications. *The Journal of clinical and aesthetic dermatology*, *10*(7), 14.

Dreno, B., Khosrotehrani, K., De Barros Silva, G., Wolf, J. R., Kerob, D., Trombetta, M., ... & Lacouture, M. (2023). The role of dermocosmetics in the management of cancer-related skin toxicities: international expert consensus. *Supportive Care in Cancer*, *31*(12), 672.

Oczkowski, M. (2021). Health-promoting effects of bioactive compounds in blackcurrant (Ribes nigrum L.) Berries. *Roczniki Państwowego Zakładu Higieny*, *72*(3), 229-238.

Kendir, G., Köroglu, A., Özkan, S., Özgen Özgacar, S., Karaoglu, T., & Gargari, S. (2016). Evaluation of antiviral and antimicrobial activities of Ribes species growing in Turkey. *Journal of Biologically Active Products from Nature*, *6*(2), 136-149.

Kupnik, K., Primožič, M., Knez, Ž., & Leitgeb, M. (2023). Trehalose. In *Valorization of Biomass to Bioproducts* (pp. 163-207). Elsevier.

Richard, S. A. (2021). Exploring the pivotal immunomodulatory and anti-inflammatory potentials of glycyrrhizic and glycyrrhetinic acids. *Mediators of Inflammation*, *2021*.

Yang, Y., Zhu, Q., Zhong, Y., Cui, X., Jiang, Z., Wu, P., ... & Zhao, S. (2020). Synthesis, anti-microbial and anti-inflammatory activities of 18β-glycyrrhetinic acid derivatives. *Bioorganic Chemistry*, *101*, 103985.

Naini, M. A., Zargari-Samadnejad, A., Mehrvarz, S., Tanideh, R., Ghorbani, M., Dehghanian, A., ... & Iraji, A. (2021). Anti-inflammatory, antioxidant, and healing-promoting effects of Aloe vera extract in the experimental colitis in rats. *Evidence-Based Complementary and Alternative Medicine*, *2021*.

Govindarajan, S., Babu, S. N., & Noor, A. (2020). Evaluation of In Vitro and In Vivo Anti-oxidant and Anti-inflammatory Potential of Aloe vera Gel Extract. In *Phytomedicine* (pp. 145-155). CRC Press.

Donato‐Trancoso, A., V. de Carvalho Faria, R., C. de S. Ribeiro, B., Nogueira, J. S., Atella, G. C., Chen, L., & Romana‐Souza, B. (2023). Dual effects of extra virgin olive oil in acute wounds. *Wound Repair and Regeneration*, *31*(3), 338-348.

Dauber, C., Parente, E., Zucca, M. P., Gámbaro, A., & Vieitez, I. (2023). Olea europea and By-Products: Extraction Methods and Cosmetic Applications. *Cosmetics*, *10*(4), 112.

Uchida, Y., & Park, K. (2021). Ceramides in skin health and disease: an update. *American Journal of Clinical Dermatology*, *22*(6), 853-866.

Kahraman, E., Kaykın, M., Şahin Bektay, H., & Güngör, S. (2019). Recent advances on topical application of ceramides to restore barrier function of skin. *Cosmetics*, *6*(3), 52.

Nădăban, A., Rousel, J., El Yachioui, D., Gooris, G. S., Beddoes, C. M., Dalgliesh, R. M., ... & Bouwstra, J. A. (2023). Effect of sphingosine and phytosphingosine ceramide ratio on lipid arrangement and barrier function in skin lipid models. *Journal of Lipid Research*, *64*(8).

Sochorová, M., Audrlická, P., Červená, M., Kováčik, A., Kopečná, M., Opálka, L., ... & Vávrová, K. (2019). Permeability and microstructure of cholesterol-depleted skin lipid membranes and human stratum corneum. *Journal of colloid and interface science*, *535*, 227-238.
