# Supplementary material for: Evaluation of the efficacy of topical cosmetic products in patients with hand-and-foot syndrome undergoing oncological treatments
Source: Oncologist. 2026 Jun 12;31(8):oyag233. doi: 10.1093/oncolo/oyag233 (PMC13351728; doi:10.1093/oncolo/oyag233)
Supplement: oyag233_Supplementary_Data [file oyag233_supplementary_data.zip › Supplement_Data/Supplementary Box S2.docx]

Original Article

**Evaluation of the efficacy of topical cosmetic products in patients with hand-and-foot syndrome (HFS) undergoing oncological treatments**

Antonia Martuscelli, MSc^1*^; Giulio Tosti, MD^2^; Patrick Maisonneuve, DiplEng^3^; Carolina Redaelli, MD^1^; Mirella Indino^4^; Martina Cereda^4^; Giuseppe Curigliano, MD, PhD^5,6^; Ida Minchella, MD^6^

^1^Oncology Aesthetics Center, IEO Istituto Europeo di Oncologia IRCCS, 20141 Milan, Italy

^2^Dermato-Oncology Unit, IEO European Institute of Oncology IRCCS, 20141 Milan, Italy

^3^Division of Epidemiology and Biostatistics, IEO European Institute of Oncology IRCCS, 20141 Milan, Italy

^4^IEO Istituto Europeo di Oncologia IRCCS, 20141 Milan, Italy

^5^Department of Oncology and Hemato-Oncology, University of Milano La Statale, 20122 Milan, Italy

^6^Division of Early Drug Development for Innovative Therapies, IEO European Institute of Oncology IRCCS, 20141 Milan, Italy

*** Corresponding author**: Antonia Martuscelli. M.Sc; Scientific coordinator at Oncology Aesthetics Center, IEO. Address: IEO Istituto Europeo di Oncologia IRCCS, via Ripamonti 435, 20141, Milan, Italy. E-mail: [a.martuscelliresearch@dermophisiologique.it](mailto:a.martuscelliresearch@dermophisiologique.it) .

**Supplementary Box S2: Diary of patient’s compliance with the product use protocol**

**PATIENT ID NUMBER ____________________ INITIAL VISIT DATE (date of receipt of the questionnaire) ___________**


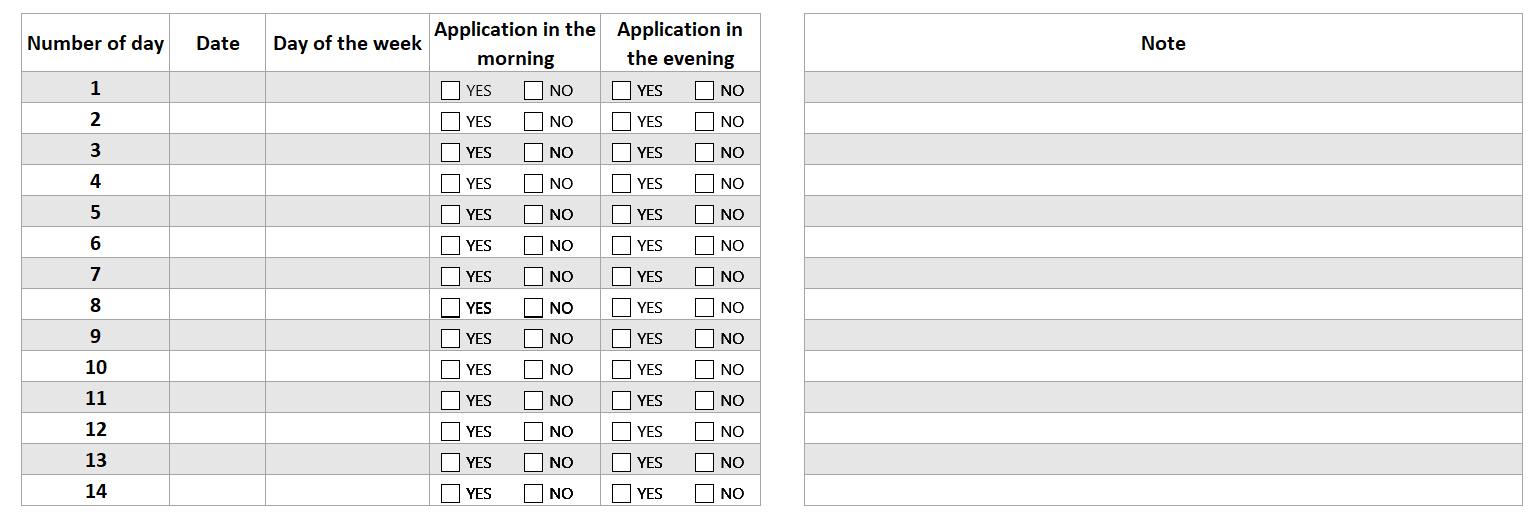

Tick ​​the box corresponding to the application of the products in the morning and in the evening, on the days indicated (YES= the products were applied; NO= the products were not applied).

In the "Notes" section, indicate any reports regarding the application of the products or your state of health.

Page 1/3

**PATIENT ID NUMBER ____________________**


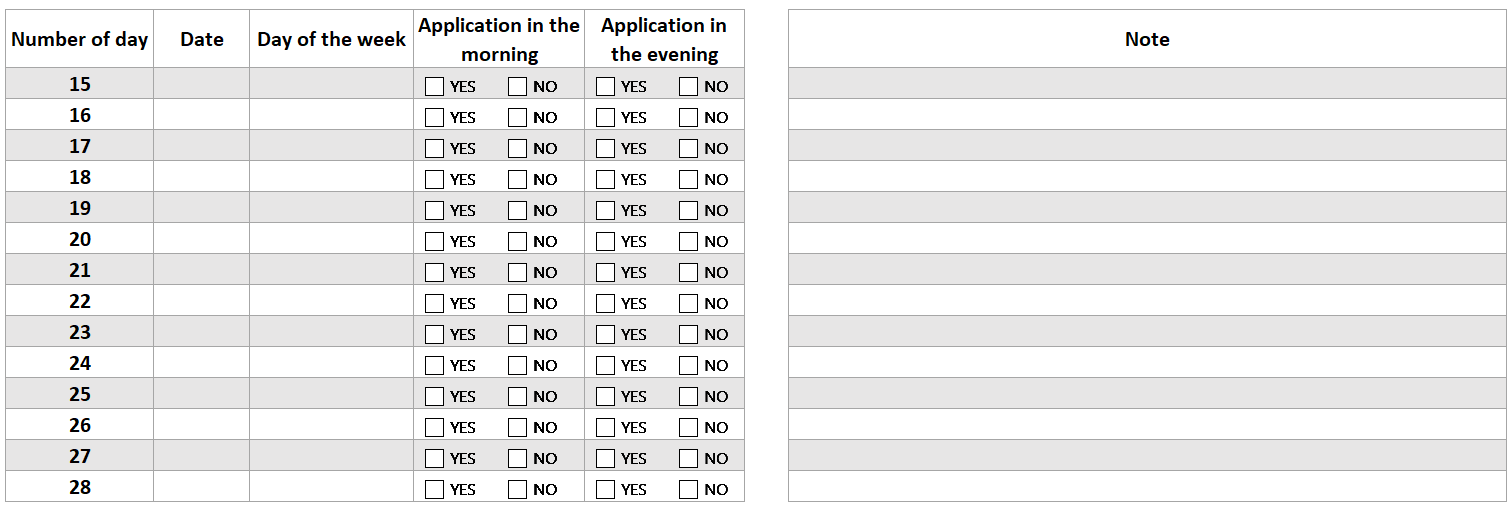


Tick ​​the box corresponding to the application of the products in the morning and in the evening, on the days indicated (YES= the products were applied; NO= the products were not applied).

In the "Notes" section, indicate any reports regarding the application of the products or your state of health.

Page 2/3

**PATIENT ID NUMBER _________________ FOLLOW-UP VISIT DATE (date of return of the questionnaire) ___________**


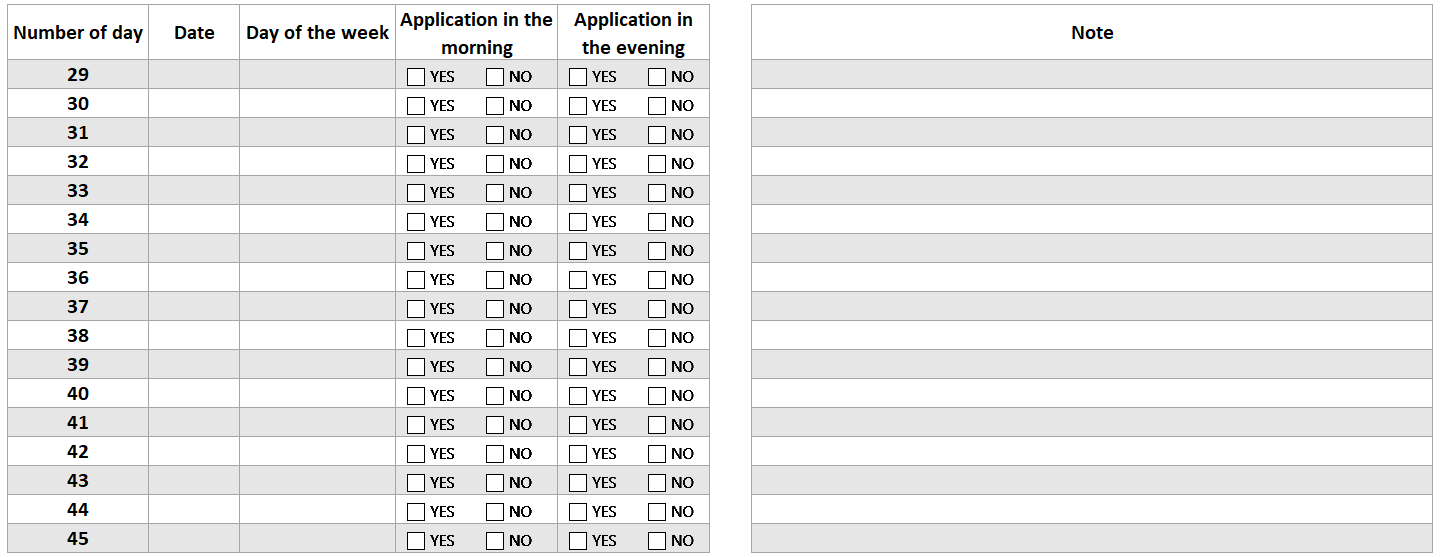


Tick ​​the box corresponding to the application of the products in the morning and in the evening, on the days indicated (YES= the products were applied; NO= the products were not applied).

In the "Notes" section, indicate any reports regarding the application of the products or your state of health.

Page 3/3
