# Supplementary material for: Evaluation of the efficacy of topical cosmetic products in patients with hand-and-foot syndrome undergoing oncological treatments
Source: Oncologist. 2026 Jun 12;31(8):oyag233. doi: 10.1093/oncolo/oyag233 (PMC13351728; doi:10.1093/oncolo/oyag233)
Supplement: oyag233_Supplementary_Data [file oyag233_supplementary_data.zip › Supplement_Data/Supplementary Table S1.docx]

Original Article

**Evaluation of the efficacy of topical cosmetic products in patients with hand-and-foot syndrome (HFS) undergoing oncological treatments**

Antonia Martuscelli, MSc^1*^; Giulio Tosti, MD^2^; Patrick Maisonneuve, DiplEng^3^; Carolina Redaelli, MD^1^; Mirella Indino^4^; Martina Cereda^4^; Giuseppe Curigliano, MD, PhD^5,6^; Ida Minchella, MD^6^

^1^Oncology Aesthetics Center, IEO Istituto Europeo di Oncologia IRCCS, 20141 Milan, Italy

^2^Dermato-Oncology Unit, IEO European Institute of Oncology IRCCS, 20141 Milan, Italy

^3^Division of Epidemiology and Biostatistics, IEO European Institute of Oncology IRCCS, 20141 Milan, Italy

^4^IEO Istituto Europeo di Oncologia IRCCS, 20141 Milan, Italy

^5^Department of Oncology and Hemato-Oncology, University of Milano La Statale, 20122 Milan, Italy

^6^Division of Early Drug Development for Innovative Therapies, IEO European Institute of Oncology IRCCS, 20141 Milan, Italy

*** Corresponding author**: Antonia Martuscelli. M.Sc; Scientific coordinator at Oncology Aesthetics Center, IEO. Address: IEO Istituto Europeo di Oncologia IRCCS, via Ripamonti 435, 20141, Milan, Italy. E-mail: [a.martuscelliresearch@dermophisiologique.it](mailto:a.martuscelliresearch@dermophisiologique.it) .

**Supplementary Table S1: Baseline measures for Skin hydration, Skin erythema and the three subscales of the Skindex-16 according patients’ characteristics and compliance**

|  | **Patients** | **Corneometer®** | **Mexameter®** | **Skindex-16** | **Skindex-16** | **Skindex-16** |
| --- | --- | --- | --- | --- | --- | --- |
|  | **number** | **Skin**  **hydration^†^** | **Skin**  **erythema^†^** | **Symptoms**  **score** | **Functioning**  **score** | **Emotions**  **score** |
|  | N | Mean ± SD (standard deviation) | Mean ± SD (standard deviation) | Mean ± SD (standard deviation) | Mean ± SD (standard deviation) | Mean ± SD (standard deviation) |
| **All patients** | 53 | 35.2 ± 11.6 | 368.7 ± 58.2 | 60.0 ± 24.0 | 43.5 ± 21.0 | 54.4 ± 20.4 |
| **Age** |  |  |  |  |  |  |
| <50 years | 8 | 30.9 ± 11.2 | 350.1 ± 82.1 | 46.9 ± 28.6 | 34.6 ± 25.4 | 43.2 ± 25.3 |
| 50-59 years | 24 | 34.5 ± 12.9 | 389.0 ± 53.6 | 66.7 ± 19.0 | 46.5 ± 23.3 | 58.6 ± 20.2 |
| ≥60 years | 21 | 37.7 ± 10.2 | 352.6 ± 47.4 | 57.3 ± 25.8 | 43.5 ± 16.0 | 53.7 ± 17.9 |
| p-value* |  | 0.34 | 0.07 | 0.10 | 0.39 | 0.18 |
| **Sex** |  |  |  |  |  |  |
| Female | 51 | 35.3 ± 11.8 | 366.8 ± 58.6 | 59.5 ± 24.3 | 43.4 ± 21.3 | 54.5 ± 20.8 |
| Male | 2 | 32.8 ± 4.6 | 416.2 ± 4.8 | 72.9 ± 8.8 | 46.7 ± 14.1 | 51.2 ± 1.7 |
| p-value* |  | 0.77 | 0.24 | 0.44 | 0.83 | 0.83 |
| **Pathology** |  |  |  |  |  |  |
| Colon cancer | 2 | 31.9 ± 1.1 | 371.9 ± 46.8 | 62.2 ± 24.5 | 45.0 ± 21.5 | 57.3 ± 17.5 |
| Breast cancer | 39 | 34.9 ± 12.8 | 373.8 ± 45.2 | 68.8 ± 8.8 | 50.0 ± 14.1 | 66.7 ± 47.1 |
| Ovarian cancer | 6 | 37.1 ± 8.1 | 293.2 ± 65.9 | 39.6 ± 32.4 | 50.0 ± 4.7 | 44.0 ± 15.2 |
| Lung cancer | 2 | 32.5 ± 12.0 | 428.9 ± 149 | 47.9 ± 26.3 | 30.6 ± 24.4 | 33.3 ± 28.1 |
| Renal cancer | 4 | 38.5 ± 9.9 | 401.1 ± 43.0 | 62.5 ± 13.2 | 42.5 ± 17.7 | 56.0 ± 5.7 |
| p-value* |  | 0.95 | **0.005** | 0.48 | 0.60 | 0.07 |
| **Therapy cycle** |  |  |  |  |  |  |
| Start | 26 | 37.0 ± 12.0 | 370.5 ± 41.8 | 65.2 ± 18.1 | 48.1 ± 14.8 | 56.0 ± 11.5 |
| Middle | 22 | 34.1 ± 12.2 | 363.6 ± 75.9 | 53.4 ± 28.1 | 36.1 ± 22.1 | 48.5 ± 25.1 |
| End | 5 | 31.3 ± 5.2 | 381.9 ± 50.1 | 61.7 ± 29.2 | 52.7 ± 35.6 | 71.4 ± 26.5 |
| p-value* |  | 0.51 | 0.80 | 0.24 | 0.08 | 0.06 |
| **Compliance ^‡^** |  |  |  |  |  |  |
| Full | 14 | 38.8 ± 12.4 | 373.5 ± 71.4 | 63.1 ± 19.6 | 43.1 ± 20.1 | 54.8 ± 19.5 |
| Intermediate | 23 | 35.0 ± 11.0 | 369.3 ± 60.9 | 53.6 ± 24.4 | 47.4 ± 22.6 | 54.9 ± 21.8 |
| Poor | 16 | 32.4 ± 11.8 | 363.6 ± 42.9 | 66.4 ± 26.0 | 38.3 ± 19.6 | 53.3 ± 20.4 |
| p-value* |  | 0.34 | 0.90 | 0.23 | 0.42 | 0.97 |

**^†^** Average of three consecutive measures at multiple sites: back of the right hand and dorsum of the right foot for hydration, right-foot plant, right-foot fingertip, right-hand palm, and right-hand fingerprint for erythema.

^‡^ Compliance: the number of applications reported by the patients in their diary: fully compliant (no omission in product application), mostly compliant (no more than 10 omissions in product application), less compliant (more than 10 omission in product application).

* p-value obtained from analysis of variance (ANOVA) for continuous variables and Fisher’s exact test for categorical variables.
