# Supplementary material for: Evaluation of the efficacy of topical cosmetic products in patients with hand-and-foot syndrome undergoing oncological treatments
Source: Oncologist. 2026 Jun 12;31(8):oyag233. doi: 10.1093/oncolo/oyag233 (PMC13351728; doi:10.1093/oncolo/oyag233)
Supplement: oyag233_Supplementary_Data [file oyag233_supplementary_data.zip › Supplement_Data/Supplementary Table S2.docx]

Original Article

**Evaluation of the efficacy of topical cosmetic products in patients with hand-and-foot syndrome (HFS) undergoing oncological treatments**

Antonia Martuscelli, MSc^1*^; Giulio Tosti, MD^2^; Patrick Maisonneuve, DiplEng^3^; Carolina Redaelli, MD^1^; Mirella Indino^4^; Martina Cereda^4^; Giuseppe Curigliano, MD, PhD^5,6^; Ida Minchella, MD^6^

^1^Oncology Aesthetics Center, IEO Istituto Europeo di Oncologia IRCCS, 20141 Milan, Italy

^2^Dermato-Oncology Unit, IEO European Institute of Oncology IRCCS, 20141 Milan, Italy

^3^Division of Epidemiology and Biostatistics, IEO European Institute of Oncology IRCCS, 20141 Milan, Italy

^4^IEO Istituto Europeo di Oncologia IRCCS, 20141 Milan, Italy

^5^Department of Oncology and Hemato-Oncology, University of Milano La Statale, 20122 Milan, Italy

^6^Division of Early Drug Development for Innovative Therapies, IEO European Institute of Oncology IRCCS, 20141 Milan, Italy

*** Corresponding author**: Antonia Martuscelli. M.Sc; Scientific coordinator at Oncology Aesthetics Center, IEO. Address: IEO Istituto Europeo di Oncologia IRCCS, via Ripamonti 435, 20141, Milan, Italy. E-mail: [a.martuscelliresearch@dermophisiologique.it](mailto:a.martuscelliresearch@dermophisiologique.it) .

**Supplementary Table S2: Clinical improvements and improvements in skin hydration, skin erythema and Skindex-16 scores after 45 days of treatments with the study products, according to baseline patients’ characteristics and compliance****.**

|  | **Patients** | **Corneometer®** | **Mexameter®** | **Skindex-16** | **Skindex-16** | **Skindex-16** | **HFS** | **Clinical** |
| --- | --- | --- | --- | --- | --- | --- | --- | --- |
|  |  | **Skin**  **hydration**^†^ | **Skin**  **erythema**^†^ | **Symptoms**  **score** | **Functioning**  **score** | **Emotions**  **score** | **Improvement^§^**  **Grade 1 to 0** | **improvement^¶^**  **(Photography)** |
|  | NUMBER | Δ (95% CI) ^‡^ | Δ (95% CI) ^‡^ | Δ (95% CI) ^‡^ | Δ (95% CI) ^‡^ | Δ (95% CI) ^‡^ | NUMBER (%) | NUMBER (%) |
| **All patients** | 53 | 33.0 (25.3 to 40.7) | -82.9 (-98.2 to -66.6) | -40.3 (-46.4 to -34.2) | -28.6 (-33.8 to -23.3) | -37.6 (-42.5 to -32.6) | 15 (28.3) | 31 (58.5) |
| **Age** |  |  |  |  |  |  |  |  |
| <50 years | 8 | 34.7 (20.4 to 49.1) | -82.9 (-114 to -52.1) | -34.9 (-52.2 to -17.6) | -27.5 (-42.7 to -12.3) | -31.8 (-51.5 to -12.2) | 4 (50.0) | 5 (62.5) |
| 50-59 years | 24 | 36.4 (24.4 to 48.5) | -90.3 (-118 to -62.7) | -46.0 (-54.9 to -37.1) | -31.3 (-41.0 to -21.5) | -41.9 (-49.4 to -34.3) | 7 (29.2) | 17 (70.8) |
| ≥60 years | 21 | 28.4 (14.5 to 42.4) | -74.6 (-97.2 to -52.0) | -35.9 (-46.5 to -25.4) | -25.9 (-32.4 to -19.4) | -34.8 (-41.8 to -27.8) | 4 (19.0) | 9 (42.9) |
| p-value* |  | 0.63 | 0.65 | 0.24 | 0.64 | 0.27 | 0.26 | 0.16 |
| **Sex** |  |  |  |  |  |  |  |  |
| Female | 51 | 34.1 (26.3 to 41.9) | -80.8 (-96.4 to -65.2) | -40.4 (-46.7 to -34.0) | -28.5 (-34.0 to -23.0) | -37.8 (-42.9 to -32.6) | 15 (29.4) | 30 (58.8) |
| Male | 2 | 5.4 (-112 to 124) | -136.5 (-140 to -133) | -39.6 (-119 to 39.8) | -30.0 (-157 to 97.1) | -32.1 (-47.3 to -17.0) | 0 ( 0.0) | 1 (50.0) |
| p-value* |  | 0.15 | 0.17 | 0.96 | 0.91 | 0.67 | 1.00 | 1.00 |
| **Pathology** |  |  |  |  |  |  |  |  |
| Colon cancer | 2 | 10.9 (-181 to 202) | -73.4 (-267 to 120) | -20.8 (-391 to 350) | 6.7 (-290 to 303) | -16.7 (-168 to 135) | 1 (50.0) | 2 (100.) |
| Breast cancer | 39 | 33.4 (24.1 to 42.7) | -87.3 (-105 to -69.7) | -42.7 (-50.2 to -35.3) | -31.1 (-36.8 to -25.4) | -40.7 (-46.1 to -35.3) | 12 (30.8) | 23 (59.0) |
| Ovarian cancer | 6 | 32.9 ( 8.4 to 57.3) | -60.9 (-101 to -21.2) | -30.6 (-44.6 to -16.5) | -19.4 (-34.0 to -4.87) | -21.4 (-42.5 to -0.40) | 1 (16.7) | 2 (33.3) |
| Lung cancer | 2 | 39.8 (-109 to 188) | 27.7 (-20.5 to 75.9) | -39.6 (-331 to 252) | -43.3 (-85.7 to -0.98) | -44.0 (-180 to 92.1) | 1 (50.0) | 1 (50.0) |
| Renal cancer | 4 | 37.2 (-24.1 to 98.4) | -133 (-161 to -105) | -41.7 (-53.8 to -29.6) | -27.5 (-62.8 to -7.78) | -38.1 (-63.8 to -12.4) | 0 ( 0.0) | 3 (75.0) |
| p-value* |  | 0.84 | **0.009** | 0.54 | **0.03** | 0.05 | 0.48 | 0.63 |
| **Therapy cycle** |  |  |  |  |  |  |  |  |
| Start | 26 | 24.4 (13.5 to 35.4) | -83.7 (-109 to -58.8) | -41.8 (-49.5 to -34.2) | -32.1 (-37.6 to -26.5) | -38.4 (-43.8 to -32.9) | 7 (26.9) | 12 (46.2) |
| Middle | 22 | 38.0 (26.2 to 49.8) | -84.3 (-108 to -61.0) | -40.7 (-51.7 to -29.7) | -26.8 (-36.0 to -17.7) | -36.8 (-46.9 to -26.7) | 7 (31.8) | 15 (68.1) |
| End | 5 | 55.6 (27.2 to 84.0) | -72.5 (-120 to -25.1) | -30.8 (-64.3 to 2.62) | -18.0 (-59.1 to 23.1) | -36.7 (-59.8 to -13.5) | 1 (20.0) | 4 (80.0) |
| p-value* |  | **0.04** | 0.91 | 0.60 | 0.28 | 0.95 | 0.91 | 0.23 |
| **Compliance** |  |  |  |  |  |  |  |  |
| Full | 14 | 31.7 (14.6 to 48.8) | -104 (-138 to -68.8) | -45.8 (-55.9 to -35.8) | -30.7 (-38.9 to -22.5) | -41.2 (-52.3 to -30.0) | 3 (21.4) | 8 (57.1) |
| Intermediate | 23 | 28.7 (17.2 to 40.2) | -71.0 (-95.9 to -46.1) | -29.7 (-38.7 to -20.7) | -28.0 (-38.3 to -17.7) | -32.4 (-39.8 to -25.0) | 7 (30.4) | 12 (52.2) |
| Poor | 16 | 40.4 (25.3 to 55.5) | -82.0 (-106 to -58.0) | -50.8 (-62.5 to -39.1) | -27.5 (-36.0 to -19.0) | -41.8 (-51.0 to -32.6) | 5 (31.3) | 11 (68.8) |
| p-value* |  | 0.43 | 0.22 | **0.006** | 0.89 | 0.19 | 0.86 | 0.60 |

† Average of three consecutive measures at multiple sites: back of the right hand and dorsum of the right foot for hydration, right-foot plant, right-foot fingertip, right-hand palm, and right-hand fingerprint for erythema.

‡ Δ Mean difference; CI Confidence intervals

§ Based on oncologist evaluation with CTCAE v. 5.0 (Common Terminology Criteria for Adverse Events);

¶ Evaluation by a dermatologist and an oncologist base on photos taken at baseline and at 45 days;

* p-value obtained from analysis of variance (ANOVA) for continuous variables and Fisher’s exact test for categorical variables.
